# Pemphigus: use of the Japanese severity index in 56 Moroccan patients

**DOI:** 10.11604/pamj.2013.16.96.2184

**Published:** 2013-11-14

**Authors:** Hakima Benchikhi, Samira Nani, Hanane Baybay, Said Amal, Fatim-Zahra Mernissi

**Affiliations:** 1 Department of Dermatology Ibn Rochd Universary Hospital, Casablanca, Morroco; 2 Laboratory of epidemiologie, Faculty of Medicine, Casablanca, Morroco; 3 Department of Dermatology, Hassan II Universary Hospital, Fes, Morroco; 4 Department of Dermatology, Ibn Tofail Universary Hospital, Marrakech, Morroco

**Keywords:** pemphigus, severity score

## Abstract

**Introduction:**

In pemphigus, there still is no consensus on parameters mesuring clinically the disease severity. The aim of this study is to use the Japanese severity index in Moroccan patients with pemphigus.

**Methods:**

Multicenter prospective study from September 2007 to September 2009 including consecutive patients with confirmed pemphigus. We used the Japenese severity index for pemphigus. For each patient, the score was calculated at diagnosis and at 6 months of follow-up and correlated to type of pemphigus; mean dosage of corticosteroids and clinical statuts at 6 months: dead or not.

**Results:**

Fifty six patients were included, 20 men and 36 women, mean age 46.62 ± 15.9 years. At diagnosis, the mean initial score was 7.7 + 2.36; at six months, it was 1.61 + 1.83. The score variation at 6 months and inclusion was 6.19 ± 2.18 for deep pemphigus and 5.43 ± 2.85 for superficial pemphigus (p = 0,3 non significant). At six months, 4 patients were dead: their initial score was 11 + 1.41 while the initial score in the 52 patients was 7.4±2.03 (p = 0,001, significant). Data showed no correlation between initial severity scores and cumulative dosage of corticosteroids at 6 months (Pearson coefficient of correlation 0.144; p = 0.580).

**Conclusion:**

In this study, initial severity scores for pemphigus were high and decreased at 6 months of treatment. Both deep pemphigus and superficial pemphigus were severe as their score variation was similar. Japenese severity score is useful for Moroccan patients with pemphigus.

## Introduction

Understanding of fundamental pathomecanisms of pemphigus showed a marked improvement in recent years [1]. However, there is still no consensus on parameters mesuring clinically the severity of disease. Thus the definition of clinical improvement and clinical evaluation of drug efficacy remain confusing. In 1993, the Japanese Ministry of welfare and health approved a draft for severity index for pemphigus [2]. This index reflects the severity of a patient's condition and further differentiates many severe cases as being pemphigus vulgaris, while the majority of moderate cases are shown to be pemphigus foliaceus [2, 3]. In 1997 then in 2000, the criteria of the index were revised [4]. In Morocco, pemphigus is a rare but severe disease and measuring the severity is useful for adapting treatment and follow-up (HB, Be). We aim to use the Japanese new criteria of severity in our pemphigus patients in order to assess its utility and its capacity to grade the severity of disease.

## Methods

From September 2007 to September 2009, a multicentric study was conducted in three departments of Dermatology in Morocco's Hassan II University Hospital, Fes; Ibn Rushd University Hospital, Casablanca, and Ibn Tofeil University Hospital, Marrakesh. All new cases of pemphigus were included and were defined by: 1) presence of recurrent blister formation, erosions and crusts; 2) histological detection of intraepidermal blistering; 3) presence of interkeratinocytes autoantibodies in cutaneous direct immunofluorescence.

Types of pemphigus were further defined as follows: 1) pemphigus vulgaris: suprabasal acantholysis, oral erosions; 2) pemphigus vegetans: vegetant bullae, suprabasal acantholysis prominent acanthosis, neutrophilic and/or eosinophilic spongiosis and pustules, 3) pemphigus foliaceus: scales, crusts and erosions predominant; subcorneal acantholysis; 4) pemphigus erythematosus, facial butterfly shaped erythema, subcorneal acantholysis; 5) pemphigus herpetiformis: vesicular erythema, eosinophilic and /or neutrophilic spongiosis. Types of pemphigus were divided in two groups: deep pemphigus (vulgaris and vegetant) and superficial (foliaceus and erythematosus).

We used the severity index for pemphigus (Table 1) as defined by the Japanese Ministry of Welfare and Health [5]. Each of the four items was scored from 0 to 3. The maximal score was 12. The total of the scores for each item gave the following severity index: total score < 5 mild; 5-7 moderate, >7 severe. For each patient, the score was calculated at the initial diagnosis and then at 6 months of follow-up. The score was correlated to the type of pemphigus; the mean dosage of corticosteroids received at 6 months and the clinical status at 6 months (dead or not).

**Table 1:** Revised severity index for pemphigus

Score	Affected area (%)^a^	Nikolsky phenomenon	Number new blisters per day	Oral lesions (%)^b^
3	<15	Distinct	<5	<30
2	5-15	Positive	1-5	5-30
1	<5	Only focal	Occasional	<5
0	None	None	None	None

Adapted from Ikeda S, Imamura S, Hashimoto I, Morioka S, et al. History of the establishment and revision criteria, severity index and therapeutic guidelines for pemphigus in Japan. Arch Dermatol Res. 2003 Apr;295 Suppl 1:S12-6. The total of the scores for each item gives the severity index.

a Percentage of affected area in relation to total skin surface area

b Percentage of affected area in relation to total mucous membrane surface area

c A few blisters per week.

Oral consent was obtained from respondents after objectives of the study were explained, and confidentiality and anonymity ensured. The agreement of ethics committee was not necessary because it gives his opinion only on intervention studies.

All data were analyzed using SPSS software. Distribution of frequency and position and dispersion measurements and calculation of proportions were performed. The Student t test was used to compare means. In the whole analysis, p < 0.05 was considered statistically significant. The correlation between initial severity scores and cumulative dosage of corticosteroids at 6 months was tested by the Pearson coefficient.

## Results

Sixty eight patients were included but clinical data was available only for 56 patients at month 6 of follow-up. Among these 56 patients, 32 were from Dermatology department of Hassan II Universary Hospital, 17 from Department of dermatology, Ibn Rushd Universary Hospital, and 7 from Department of dermatology, Ibn Tofeil Universary Hospital. There were 20 men and 36 women, mean age 46.62 ± 15.9 years (extremes 19 and 80). The types of pemphigus were as follows: 35 pemphigus vulgaris, 3 pemphigus vegetans, 10 pemphigus erythematosus, 4 pemphigus foliaceus, 4 pemphigus herpetiformis.

At diagnosis, the initial score was 7.7 ± 2.36. At six months, it was 1.61 ± 1.83 (p < 0,001). Table 2 shows initial score, 6-month score and its variation according to the type of pemphigus. At six months, 4 patients were dea: their initial score was 11±1,41 while the initial score in the 52 patients alive at 6 months was 7,4±2,03 (p = 0,001, significant). Data shows no correlation between initial severity scores and cumulative dosage of corticosteroids at 6 months (Pearson coefficient of correlation 0.144; p = 0.580).

**Table 2:** Initial severity score, 6-month score and its variation according to the clinical form in 52 patients with pemphigus

	Initial score (mean)	6-month score (mean)	Variation of scores
Deep pemphigus (n = 38)	7,97 + 2,24	1,69 + 2,02	6,19 ± 2,18
Superficial pemphigus (n = 14)†	7, 18 + 1,93	1,75 + 2,32	5,43 ± 2,85
p-value	0.23	0.92	0.30

† 4 pemphigus herpetiformis excluded.

## Discussion

We have used this method of scoring in pemphigus patients and these results have shown that initial high scores are correlated with a poor status at 6 months; both deep pemphigus and superficial pemphigus were severe as their score variations were similar.

The reasons for choosing this particular score are multiple. This score has been established after nationwide epidemiologic studies in Japan. We didn't use the first version because it included the presence of dosage of auto antibodies, and blood test could have been necessary [2, 3]. In the latest version, this criterion was cancelled [4]. We found that two criteria were easy to calculate: 1) the affected surface area using the rule of Wallace as for burns; 2) the number of new blisters per day. However, the evaluation of the percentage of affected oral area in relation to total mucous membrane area was more subjectively performed. The most difficult to evaluate was the Nikolsky phenomenon as distinguished by the index: it could be absent, only focal, positive or distinct. We found that these two latest definitions are similar, knowing that the score can vary from one point, which is 12% of the maximum score. Nevertheless, the Nikolsky sign is important for evaluating clinically the severity of pemphigus [6].

In a another score performed by Agorwal and called PAAS, the Nikolsky sign was defined as absent, or present perilesionally or at a distant site [7]. This type of description is more likely to be used. However, the complexity of the PAAS makes it difficult to use in a practical manner.

Despite these constraints, we found that initial scores in our patients reflected the severity of the pemphigus (more than 7) and decreased dramatically at month 6 when treatment was performed. So, as in the Japanese patients with pemphigus, the index reflected the severity of the condition in a particular patient [2, 3]. Paradoxically, although there is no validated severity index in pemphigus, the clinical severity of the disease is taken into account for increasing the intensity of the treatment in clinical therapeutic trials in pemphigus. In the study of Shaih, 73% of patients who presented with severe disease did receive additional oral corticosteroids between treatment pulses of corticosteroids and cyclophosphamide [8]. But severity was not objectively determined. In other trials, pemphigus is said “active” or “inactive” before including patients in different groups of therapy [9].

We found that higher initial scores were correlated to severe prognosis in patients reflecting the severity of the disease as in the Japanese study [2, 3]. However, as our pemphigus are severe condition at diagnosis, no correlation between dosage of corticosteroids and initial severity scores was found. All our patients presented with extremely severe condition and received high dosage of corticosteroids associated when available, with azathioprine or cyclophosphamide pulses.

Concerning the clinical type of pemphigus, we found that there were no significant differences between initial scores in deep pemphigus versus superficial pemphigus. Indeed, their variation index at diagnosis and 6-month of follow-up was similar. This data suggests that both deep and superficial pemphigus are severe condition in Moroccan patients and this was previously shown [10]. In contrast, in the Japenese studies, many severe cases were pemphigus vulgaris, while the majority of moderate cases are shown to be pemphigus foliaceus [2, 3].

## Conclusion

According to our results, the Japanese severity index for pemphigus is adapted for Moroccan patients with pemphigus. It reflected the severity of a patient's condition, and higher score was correlated with bad prognosis. Larger use will further allow us to better evaluate our patients with pemphigus.
